# B-cell depletion therapy in patients with diffuse systemic sclerosis associates with a significant decrease in PDGFR expression and activation in spindle-like cells in the skin

**DOI:** 10.1186/ar3879

**Published:** 2012-06-14

**Authors:** Dimitrios Daoussis, Athanassios C Tsamandas, Stamatis-Nick C Liossis, Ioannis Antonopoulos, Elli Karatza, Georgios Yiannopoulos, Andrew P Andonopoulos

**Affiliations:** 1 Division of Rheumatology, Department of Internal Medicine, Patras University Hospital, University of Patras Medical School, Patras, Rion, 26504, Greece; 2 Department of Pathology, Patras University Hospital, University of Patras Medical School, Patras, Rion, 26504, Greece

## Abstract

**Introduction:**

Recently, several studies assessing the clinical efficacy of rituximab (RTX) in systemic sclerosis (SSc) have reported encouraging results. We aimed at exploring whether RTX exerts its beneficial effects on fibrosis through attenuation of platelet-derived growth factor receptor (PDGFR) pathway activation.

**Methods:**

We immunohistochemically assessed skin biopsies obtained from eight patients with SSc prior to and 6 months following RTX treatment, three control SSc patients (at the same time points) and three healthy subjects. We assessed the expression of platelet-derived growth factor, PDGFR and phosphorylated (activated) PDGFR.

**Results:**

We found a strong correlation of PDGFRα and PDGFRβ expression on spindle-like cells and collagen deposition in SSc biopsies (*r *= 0.97 and *r *= 0.96 for PDGFRα and PDGFRβ, respectively; *P *< 0.0001 for both), indicating a strong link between PDGFR expression and fibrosis. Expression of PDGFRα and PDGFRβ in the papillary dermis significantly decreased following RTX administration (mean ± standard error of the mean at baseline vs. 6 months, respectively: PDGFRα, 42.05 ± 5.03 vs. 26.85 ± 3.00, *P *= 0.004; and PDGFRβ, 37.14 ± 4.94 vs. 24.01 ± 3.27, *P *= 0.012). Similarly, expression of phosphorylated PDGFRα and PDGFRβ in the papillary dermis significantly decreased following RTX administration (*P *= 0.006 and *P *= 0.013 for phospho-PDGFRα and phospho-PDGFRβ, respectively). No changes in platelet-derived growth factor tissue expression or serum levels were found following RTX treatment.

**Conclusion:**

RTX may favorably affect skin fibrosis through attenuation of PDGFR expression and activation, a finding that supports a disease-modifying role of RTX in SSc. Large-scale, multicenter studies are needed to further explore the efficacy of RTX in SSc.

## Introduction

Systemic sclerosis (SSc) represents one of the most challenging and difficult to treat diseases for rheumatologists. During recent years there has been significant progress towards a better understanding of the complex pathogenesis of the disease; however, this progress has not been translated into novel therapies. Treatment options for patients with SSc are so far limited, while no therapy has definitely shown disease-modifying properties. During the last few years, four studies - including one from our research group - have assessed the potential clinical efficacy of rituximab (RTX) in patients with SSc and have reported encouraging results [1-4]. The rationale for the use of RTX in SSc is based on strong experimental data suggesting a key role for B cells in regulating the fibrotic process [5-9]. Patients treated with RTX showed either an improvement of skin fibrosis or lung function or remained clinically stable; it should be noted, however, that stabilization alone should be considered a positive outcome in this devastating disease where patients tend to gradually worsen over time. In three of those studies where skin biopsies were performed, a significant histologic improvement in terms of reduction of collagen deposition and myofibroblast score was reported [1,3,4], suggesting a potential disease-modifying role of RTX in skin fibrosis.

RTX is a monoclonal antibody that targets and depletes B cells but it is not known how this drug may mediate its beneficial effect on fibrosis. Platelet-derived growth factor (PDGF) is a pivotal mediator of fibrosis; it has stimulatory effects on scleroderma fibroblasts by enhancing their proliferation and increasing collagen production [10]. PDGF signals through two structurally similar tyrosine kinase receptors, platelet-derived growth factor receptor (PDGFR)α and PDGFRβ. PDGF leads to homodimerization or heterodimerization of these receptors and to phosphorylation of specific tyrosine residues. In animal models, enhancement of PDGF signaling leads to fibrosis [11,12]. Moreover agonistic autoantibodies against PDGFR have been found in patients with SSc; these stimulatory autoantibodies have been suggested to participate in disease pathogenesis [13].

Based on these data, we aimed to explore whether RTX exerts its beneficial effects on fibrosis through attenuation of PDFGR pathway activation. We therefore immunohistochemically assessed the expression of PDGF, PDGFR and phospho-PDGFR, which represents the phosphorylated and therefore active form of the receptor. We report herein imunohistochemical evidence that RTX treatment associates with a significant decrease in PDGFR expression and activation in spindle-like cells in the skin, indicating that RTX may favorably affect fibrosis through modulation of PDGFR expression/activation.

## Materials and methods

Eight patients with diffuse SSc fulfilling the preliminary American College of Rheumatology criteria for the classification of the disease [14] were enrolled. Demographic and clinical characteristics of these patients have been previously reported [1,15]. A local ethics committee approved the study protocol (which fulfilled the Declaration of Helsinki requirements) and written informed consent was obtained from all participating individuals.

### Skin histology

Patients were subjected to skin biopsies (a 5 mm punch biopsy) prior to and 6 months following RTX administration (consisting of four pulses of RTX (375 mg/m^2^) weekly). Biopsies were taken from lesional skin in the forearm; the second biopsy was taken from lesional skin adjacent to (always < 2 cm) the site of the baseline biopsy. Skin biopsies were also performed in the same way and at the same time points in three SSc patients who did not receive RTX therapy (two patients treated with cyclophosphamide and one receiving no treatment). Skin biopsies from three healthy subjects were used as controls. All skin biopsies were fixed in 10% neutral buffered formalin and embedded in paraffin.

Collagen deposition was assessed and quantified using computerized image analysis as previously described [1]. Myofibroblasts (alpha smooth muscle actin-positive cells) were detected by a standard streptavidin-biotin peroxidase method using an anti-alpha smooth muscle actin antibody (Novocastra, Newcastle, UK). For the detection of PDGF-AA, PDGF-BB, PDGFRα, PDGFRβ, phospho-PDGFRα and phospho-PDGFRβ we employed a standard streptavidin-biotin peroxidase method (DAKO Cytomation Envision™ Peroxidase DAB;DAKO, Glostrup, Denmark), using the following antibodies: anti-PDGF-AA (Millipore, Billerica, Massachusetts, USA), anti-PDGF-BB (Acris, Herford, Germany), anti-PDGFRα and anti-PDGFRβ (both from Santa-Cruz Biotechnology, Santa-Cruz, California, USA), anti-phospho-PDFRα (phosphoY754; Abcam, Cambridge, UK), and anti-phospho-PDFRβ (phospho Y579; Abcam). The same staining procedure was performed on paraffin sections from human hepatocellular carcinoma, which served as a positive control for PDGFR and phospho-PDGFR staining.

PDGFR and phospho-PDFR were mainly expressed in spindle-like cells resembling fibroblasts, so we employed a high-precision image analysis system (Image-Pro Plus software program; Media Cybernetics, Silver Spring, MD, USA) to quantify the intensity of immunohistochemical staining in these cells, as previously described [16-18]. We used a color camera (KY-F55MD; Olympus, Tokyo, Japan) mounted on an Olympus BX 41F microscope. Each time, before measurements of the images were taken, we standardized the contrast and brightness and calibrated the measurement system with appropriate slides. The latter included the slides that we used as positive controls in the immunohistochemical stains, as were described above. We excluded the edges of the sections from the evaluation.

For quantitation of immunostaining intensities, we measured the inverse mean density based on the RGB color parameter. The circle profile tool of the Image-Pro Plus program was used for measurements of immunostaining of PDGFR and phospho-PDGFR in spindle-like cells. According to previous reports [16,17], in each case 20 randomly selected color video images of 512 × 512 pixels with a resolution of 0.4348 μm were performed. Measurements were made on a total of 100 cells per specimen (5 cells/image ×20). Measurements were carried out separately for spindle-like cells in the upper and lower dermis. For each set of measurements, a curve was made according to immunostaining intensity. The units arbitrarily ranged from 0 (intensity absent) to 230 (top intensity). In all immunohistochemical procedures, for negative-control purposes, the same techniques were used on tissue sections in which 1% BSA in PBS substituted the primary antibody in each case. All biopsies were evaluated simultaneously in a blinded (to treatment and date) fashion. Serum levels of PDGF-AA and PDGF-BB were measured by employing ELISA methodology using commercially available kits (both from R&D Systems, Minneapolis, MN, USA).

### Statistical analysis

Statistical analysis was performed using the GraphPad Prism software version 5 (GraphPad Software, La Jolla, CA, USA). Pearson correlation and a paired *t *test were used as indicated.

## Results

Clinical and histological outcomes following RTX treatment have been reported previously [1,15]. Briefly, we found a significant improvement of skin thickening, as clinically assessed, which coincided with histologic improvement (reduction of collagen deposition and myofibroblast score) in six out of eight patients. Histologic improvement was associated with depletion of skin-infiltrating B cells.

### PDGFR expression on spindle-like cells in scleroderma skin is strongly associated with collagen deposition and attenuates following rituximab administration

We first studied, by immunohistochemistry, the expression of PDGFRα and PDGFRβ in scleroderma versus normal skin. Expression of PDGFRα and PDGFRβ could not be detected in spindle-like cells in the dermis of normal skin. A modest expression could be detected, however, in vessels and adnexae. On the contrary, PDGFRα and PDGFRβ were highly expressed in scleroderma skin. Expression of PDGFRα and PDGFRβ was detected mainly in spindle-like cells resembling fibroblasts, in vessels and in adnexae.

**Figure 1:**
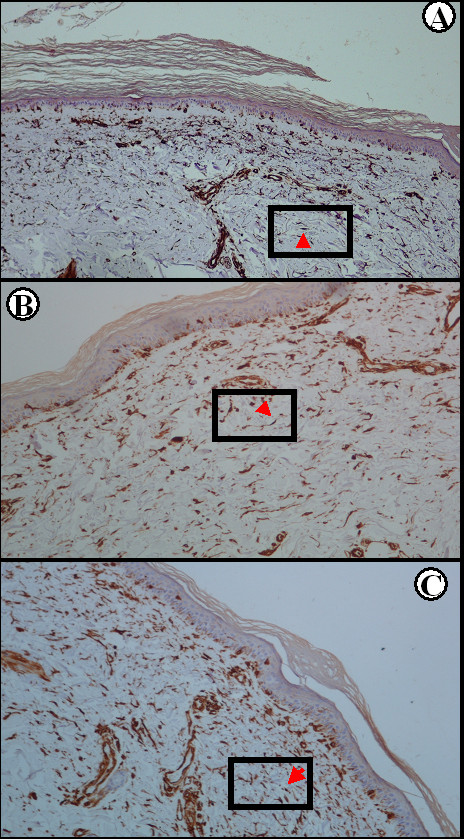
**Correlation between alpha smooth muscle actin and platelet-derived growth factor receptor expression**. Immunohistochemical expression of **(A) **alpha smooth muscle actin (αSMA), **(B) **platelet-derived growth factor receptor (PDGFR)α and **(C) **PDGFRβ in spindle-like cells in Patient 5 prior to rituximab treatment (streptavidin-biotin peroxidase ×100). Red arrow in the boxed area points to a spindle-like cell (among others) expressing αSMA, PDGFRα and PDGFRβ.

We found a strong correlation of PDGFRα and PDGFRβ expression on spindle-like cells and collagen deposition in SSc biopsies (*r *= 0.97 and *r *= 0.96 for PDGFRα and PDGFRβ, respectively; *P *< 0.0001 for both), indicating a strong link between PDGFR expression and fibrosis. We also found a strong correlation between alpha smooth muscle actin and PDGFR expression (*r *= 0.95, *P *= 0.0002 and *r *= 0.96, *P *= 0.0003 for PDGFRα and PDGFRβ, respectively) as shown in Figure 1, indicating that the majority of spindle-like cells were probably myofibroblasts.

**Figure 2:**
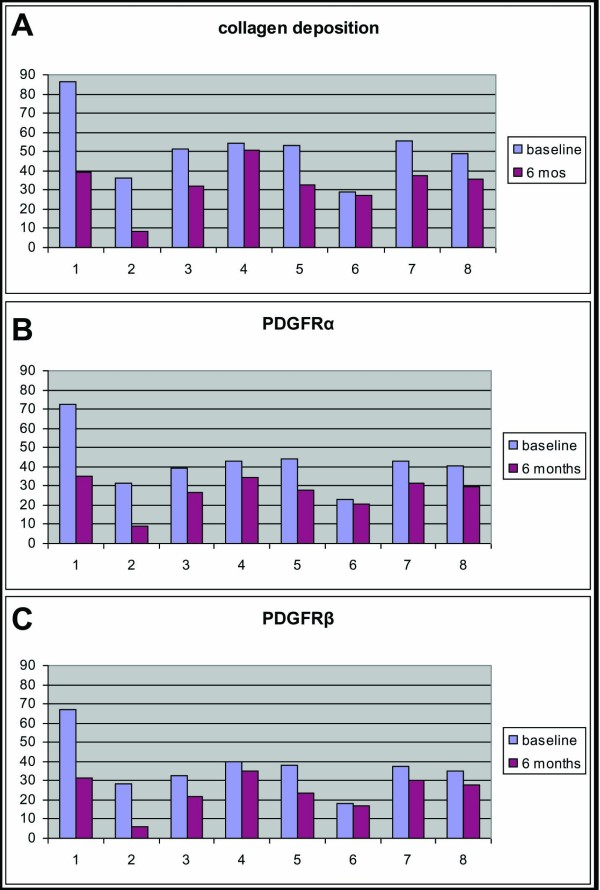
**Platelet-derived growth factor receptor expression and collagen deposition**. Rituximab (RTX)-induced resolution of skin fibrosis associates with attenuation of platelet-derived growth factor receptor (PDGFR) expression. RTX treatment mediates a significant decrease of **(B) **PDGFRα and **(C) **PDGFRβ expression on spindle-like cells in the papillary dermis, which associates with attenuation of collagen deposition **(A)**. No change in both PDGFR expression and collagen deposition was found in Patients 4 and 6, indicating a strong link between PDGFR expression and fibrosis.

Expression of PDGFRα and PDGFRβ in the papillary dermis significantly decreased following RTX administration (mean ± standard error of the mean (SEM) at baseline vs. 6 months, respectively: PDGFRα, 42.05 ± 5.03 vs. 26.85 ± 3.00, *P *= 0.004; and PDGFRβ, 37.14 ± 4.94 vs. 24.01 ± 3.27, *P *= 0.012). More specifically, expression of PDGFRα and PDGFRβ decreased in all six patients who showed histologic improvement but remained unchanged in the two patients who did not improve histologically, as shown in Figure 2. To explore whether the decrease in PDGFR expression reported above is a specific effect of RTX treatment, we studied skin biopsies from three control patients with SSc. In these three patients we found no change in the expression of PDGFRα and PDGFRβ at 6 months in the papillary dermis compared with baseline (mean ± SEM at baseline vs. 6 months, respectively: PDGFRα, 35.77 ± 6.88 vs. 35.87 ± 3.69; and PDGFRβ, 30.83 ± 6.85 vs. 33.17 ± 4.11; *P *= not significant in both cases). No changes in PDGFR expression in the reticular dermis at baseline versus 6 months was depicted in either the RTX group or the control group.

PDGFR staining in vessels and adnexae was not altered following RTX treatment, indicating that the effect of B-cell depletion therapy was specific to spindle-like cells.

The above data indicate that RTX mediates a significant attenuation of PDGFRα and PDGFRβ expression on spindle-like cells in the papillary dermis of patients with SSc.

### Expression of phosphorylated (activated) PDGFR on spindle-like cells in scleroderma skin decreases following rituximab treatment

We next studied the expression of phosphorylated PDGFRα and PDGFRβ, which represent the activated forms of the receptor. Staining patterns for phospho-PDGFRα and phospho-PDGFRβ were the same as for PDGFRα and PDGFRβ. Expression of phosphorylated PDGFRα and PDGFRβ could not be detected in spindle-like cells in the dermis of normal skin; on the contrary, these receptors were highly expressed on spindle-like cells in scleroderma skin.

**Figure 3:**
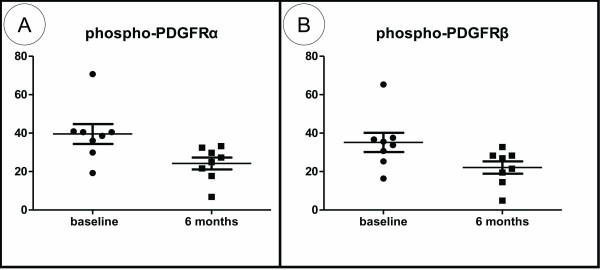
**Expression of phosphorylated platelet-derived growth factor receptor in the papillary dermis following rituximab administration**. Rituximab (RTX) mediates a significant attenuation in platelet-derived growth factor receptor (PDGFR) phosphorylation (activation) status. **(A) **Phospho-PDGFRα and **(B) **phospho-PDGFRβ expression is decreased following RTX administration.

**Figure 4:**
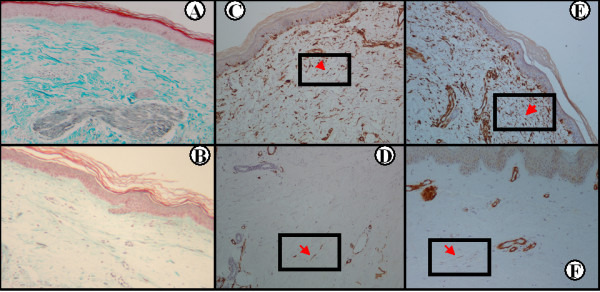
**Phosphorylated platelet-derived growth factor receptor expression in the rituximab treated systemic sclerosis patients**. Substantial reduction of collagen accumulation following rituximab (RTX) treatment **(B) **compared with baseline **(A) **in Patient 2 (Masson's trichrome ×100). Immunohistochemical staining for phosphorylated platelet-derived growth factor receptor (PDGFR)α and PDGFRβ in the same patient prior to (**(C) **and **(E)**, respectively) and following (**(D) **and **(F)**, respectively) RTX treatment, where a significant decrease in phospho-PDGFR staining can be seen (streptavidin peroxidase ×100). Red arrow in the boxed area points to a spindle-like cell (among others) expressing phosphorylated PDGFRα and PDGFRβ.

**Figure 5:**
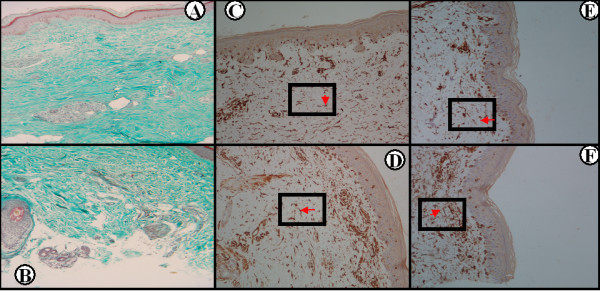
**Phosphorylated platelet-derived growth factor receptor expression in the control systemic sclerosis patients**. Collagen accumulation does not improve in a control patient following cyclophosphamide treatment **(B) **compared with baseline **(A)**. Immunohistochemical staining for phosphorylated platelet-derived growth factor receptor (PDGFR)α and PDGFRβ in the same patient prior to (**(C) **and **(E)**, respectively) and following (**(D) **and **(F)**, respectively) rituximab (RTX) treatment, where no change in phospho-PDGFR staining can be seen (streptavidin peroxidase ×100). Red arrow in the boxed area points to a spindle-like cell (among others) expressing phosphorylated PDGFRα and PDGFRβ.

Expression of phosphorylated PDGFRα and PDGFRβ in the papillary dermis significantly decreased following RTX administration (mean ± SEM at baseline vs. 6 months, respectively: phospho-PDGFRα, 39.55 ± 5.16 vs. 24.21 ± 3.11, *P *= 0.006; and phospho-PDGFRβ, 35.18 ± 4.97 vs. 22.09 ± 3.20, *P *= 0.013), as shown in Figure 3. Representative histology is presented in Figure 4. On the contrary, we found no change in the expression of phosphorylated PDGFRα and PDGFRβ at 6 months compared with baseline in the control SSc patients (mean ± SEM at baseline vs. 6 months, respectively: phospho-PDGFRα, 33.27 ± 7.21 vs. 33.63 ± 3.58; and phospho-PDGFRβ, 28.90 ± 6.92 vs. 31.07 ± 4.17; *P *= not significant in both cases). Representative histology is presented in Figure 5. Images depicting phospho-PDGFRβ staining from all study subjects can be found in Additional Files 1 to 8 (RTX-treated patients) and Additional Files 9 to 11 (control patients).

We next calculated the phospho-PDGFRα/PDGFRα ratio and the phospho-PDGFRβ/PDGFRβ ratio to assess the phosphorylation (activation) status of the receptor. We found that PDGFR on spindle-like cells is highly phosphorylated in scleroderma skin, in sharp contrast to normal skin where PDGFR and phospho-PDGFR are virtually undetectable (mean ± SEM: 0.89 ± 0.02 and 0.93 ± 0.02 for phospho-PDGFRα/PDGFRα and phospho-PDGFRβ/PDGFRβ, respectively). The ratios of phospho-PDGFRα/PDGFRα and phospho-PDGFRβ/PDGFRβ modestly declined following treatment with RTX; statistical significance was reached only for phospho-PDGFRβ/PDGFRβ (mean ± SEM at baseline vs. 6 months, respectively: phospho-PDGFRα/PDGFRα ratio, 0.92 ± 0.017 vs. 0.87 ± 0.02, *P *= 0.09; and phospho-PDGFRβ/PDGFRβ ratio, 0.93 ± 0.009 vs. 0.89 ± 0.019, *P *= 0.04).

The above data taken together indicate that RTX treatment not only mediates a significant attenuation of PDGFRα and PDGFRβ expression on spindle-like cells, but also associates with a decrease in PDGFRβ phosphorylation (activation) status.

### Serum levels and tissue expression of PDGF is not altered by rituximab treatment

We next assessed whether the attenuation of PDGFR expression and activation reported above is mediated by changes in serum levels or tissue expression of PDGF. Serum levels of PDGF-AA and PDGF-BB did not change following RTX treatment (mean ± SEM optical density at baseline vs. 6 months, respectively: PDGF-AA, 0.68 ± 0.06 vs. 0.81 ± 0.09; and PDGF-BB, 0.32 ± 0.03 vs. 0.38 ± 0.09; *P *= not significant in both cases). Similarly, we found no changes in the expression of PDGF in the papillary dermis following RTX treatment (mean ± SEM at baseline vs. 6 months, respectively: PDGF-AA, 44.61 ± 3.63 vs. 47.06 ± 3.27; and PDGF-BB, 43.84 ± 5.44 vs. 44.59 ± 2.61; *P *= not significant in both cases).

These data suggest that the attenuation of PDGFR expression/activation in scleroderma skin, following RTX treatment, is not mediated by changes of its ligand in either peripheral blood or skin.

## Discussion

We report herein immunohistochemical evidence that RTX treatment in patients with SSc associates with a significant downregulation of PDGFRα and PDGFRβ expression on spindle-like cells in the skin and with attenuation of the PDGFRβ phosphorylation (activation) status. The biologic effect of RTX on PDGFR is noteworthy since PDGF and transforming growth factor beta are considered master regulators of the fibrotic process. An animal model with mutations in PDGFRα leading to excessive kinase activity was created recently. Interestingly, this animal model is characterized by a multiorgan fibrotic disease that has striking similarities with SSc [19]. Moreover, irradiation-induced lung fibrosis in mice associates with a significant increase in PDGFR activation; blocking PDGFR signaling by specific tyrosine kinase inhibitors attenuates fibrosis and increases survival in these mice [20]. Blockade of PDGFR signaling is also effective in other models, such as the bleomycin-induced mouse model [21] and the vanadium pentoxide-induced mouse model [22] of lung fibrosis, providing evidence for the pivotal role of this pathway. The central role of PDGFR signaling in fibrosis is further supported by the reports of the antifibrotic properties of imatinib mesylate, a kinase inhibitor that partially acts through PDGFRβ inhibition, in animal models [23]. In patients with SSc, PDGF is highly expressed in the skin [24], in bronchoalveolar lavage fluid [25] as well as in peripheral blood [26].

In this study we found that PDGFR is highly phosphorylated (activated) in scleroderma skin, in sharp contrast to normal skin where phosphorylated PDGFR was undetectable. This is the first study to report the expression of the phosphorylated form of PDGFR in SSc; other studies have assessed the expression of PDGFR using antibodies not specific for the phosphorylated form of the receptor [27-29]. The strong correlation between PDGFR expression and collagen accumulation found in our study underscores the significant role of PDGFR in fibrosis. RTX induces a significant decrease in PDGFR expression and collagen accumulation. This effect seemed to be specific for RTX since PDGFR expression was not decreased in the control biopsies.

This study does not answer the question of how RTX affects the expression of PDGFR. One may hypothesize that RTX may act through elimination of agonistic anti-PDGFR autoantibodies. A direct answer to this question could be given by measurement of these autoantibodies in serum, prior to and following RTX administration, but measurement of these autoantibodies can only be made using complex and technically challenging bioassays. Nevertheless, if RTX mediates its beneficial effect on fibrosis through elimination of stimulatory autoantibodies, one would expect a more robust downregulation of PDGFRα and PDGFRβ phosphorylation (activation) status; on the contrary, we found a significant decrease in PDGFRα and PDGFRβ expression and only a modest attenuation of PDGFRβ phosphorylation. We therefore propose that other mechanisms may apply; RTX not only affects B cells but has a broader effect on the immune system.

## Conclusions

Data regarding the clinical efficacy of RTX in SSc are encouraging. The Rituximab Group of the EULAR Scleroderma Trials and Research Group reported recently that B-cell depletion therapy favorably affects skin fibrosis (data derived from 72 SSc patients treated with RTX from 27 EULAR Scleroderma Trials and Research Group centers) [30]. The data reported herein suggest that RTX may favorably affect skin fibrosis through attenuation of PDGFR expression and activation, and add further evidence in support of a disease-modifying role for RTX in SSc. A large-scale, randomized controlled study assessing the efficacy of RTX in SSc is therefore needed.

## Abbreviations

BSA: bovine serum albumin; ELISA: enzyme-linked immunosorbent assay; PBS: phosphate-buffered saline; PDGF: platelet-derived growth factor; PDGFR: platelet-derived growth factor receptor; RTX: rituximab; SEM: standard error of the mean; SSc: systemic sclerosis.

## Competing interests

The authors declare that they have no competing interests.

## Authors' contributions

DD performed the skin biopsies, analyzed the data, conceived the idea of the study and drafted the manuscript. ACT carried out the immunohistochemical assessment. S-NCL participated in data analysis, patient recruitment, study design and manuscript drafting. IA participated in data analysis and performed the ELISA. EK carried out the immunohistochemistry. GY participated in patient recruitment, study design and manuscript drafting. APA participated in data analysis, patient recruitment, study design and manuscript drafting. All authors read and approved the final manuscript.

## Supplementary Material

Additional file 1 **Phosphorylated PDGFRβ expression in skin biopsies from Patient 1 (rituximab treated)**. A and B, biopsy taken at baseline; C and D, biopsy taken at 6 months. B and D, higher magnification of the areas included in the boxes in A and C, respectively. Red arrows indicate presence of spindle-like cells that express phospho-PDGFRβ. Streptavidin-biotin peroxidase: A and C, ×100; B and D, ×400. Click here for file

Additional file 2 **Phosphorylated PDGFRβ expression in skin biopsies from Patient 2 (rituximab treated)**. A and B, biopsy taken at baseline; C and D, biopsy taken at 6 months. B and D, higher magnification of the areas included in the boxes in A and C, respectively. Red arrows indicate presence of spindle-like cells that express phospho-PDGFRβ. Streptavidin-biotin peroxidase: A and C, ×100; B and D, ×400. Click here for file

Additional file 3 **Phosphorylated PDGFRβ expression in skin biopsies from Patient 3 (rituximab treated)**. A and B, biopsy taken at baseline; C and D, biopsy taken at 6 months. B and D, higher magnification of the areas included in the boxes in A and C, respectively. Red arrows indicate presence of spindle-like cells that express phospho-PDGFRβ. Streptavidin-biotin peroxidase: A and C, ×100; B and D, ×400. Click here for file

Additional file 4 **Phosphorylated PDGFRβ expression in skin biopsies from Patient 4 (rituximab treated)**. A and B, biopsy taken at baseline; C and D, biopsy taken at 6 months. B and D, higher magnification of the areas included in the boxes in A and C, respectively. Red arrows indicate presence of spindle-like cells that express phospho-PDGFRβ. Streptavidin-biotin peroxidase: A and C, ×100; B and D, ×400. Click here for file

Additional file 5 **Phosphorylated PDGFRβ expression in skin biopsies from Patient 5 (rituximab treated)**. A and B, biopsy taken at baseline; C and D, biopsy taken at 6 months. B and D, higher magnification of the areas included in the boxes in A and C, respectively. Red arrows indicate presence of spindle-like cells that express phospho-PDGFRβ. Streptavidin-biotin peroxidase: A and C, ×100; B and D, ×400. Click here for file

Additional file 6 **Phosphorylated PDGFRβ expression in skin biopsies from Patient 6 (rituximab treated)**. A and B, biopsy taken at baseline; C and D, biopsy taken at 6 months. B and D, higher magnification of the areas included in the boxes in A and C, respectively. Red arrows indicate presence of spindle-like cells that express phospho-PDGFRβ. Streptavidin-biotin peroxidase: A and C, ×100; B and D, ×400. Click here for file

Additional file 7 **Phosphorylated PDGFRβ expression in skin biopsies from Patient 7 (rituximab treated)**. A and B, biopsy taken at baseline; C and D, biopsy taken at 6 months. B and D, higher magnification of the areas included in the boxes in A and C, respectively. Red arrows indicate presence of spindle-like cells that express phospho-PDGFRβ. Streptavidin-biotin peroxidase: A and C, ×100; B and D, ×400. Click here for file

Additional file 8 **Phosphorylated PDGFRβ expression in skin biopsies from Patient 8 (rituximab treated)**. A and B, biopsy taken at baseline; C and D, biopsy taken at 6 months. B and D, higher magnification of the areas included in the boxes in A and C, respectively. Red arrows indicate presence of spindle-like cells that express phospho-PDGFRβ. Streptavidin-biotin peroxidase: A and C, ×100; B and D, ×400. Click here for file

Additional file 9 **Phosphorylated PDGFRβ expression in skin biopsies from control Patient 1**. A and B, biopsy taken at baseline; C and D, biopsy taken at 6 months. B and D, higher magnification of the areas included in the boxes in A and C, respectively. Red arrows indicate presence of spindle-like cells that express phospho-PDGFRβ. Streptavidin-biotin peroxidase: A and C, ×100; B and D, ×400. Click here for file

Additional file 10 **Phosphorylated PDGFRβ expression in skin biopsies from control Patient 2**. A and B, biopsy taken at baseline; C and D, biopsy taken at 6 months. B and D, higher magnification of the areas included in the boxes in A and C, respectively. Red arrows indicate presence of spindle-like cells that express phospho-PDGFRβ. Streptavidin-biotin peroxidase: A and C, ×100; B and D, ×400. Click here for file

Additional file 11 **Phosphorylated PDGFRβ expression in skin biopsies from control Patient 3**. A and B, biopsy taken at baseline; C and D, biopsy taken at 6 months. B and D, higher magnification of the areas included in the boxes in A and C, respectively. Red arrows indicate presence of spindle-like cells that express phospho-PDGFRβ. Streptavidin-biotin peroxidase: A and C, ×100; B and D, ×400. Click here for file
