# Supplementary figures and images for: B-cell depletion therapy in patients with diffuse systemic sclerosis associates with a significant decrease in PDGFR expression and activation in spindle-like cells in the skin
Source: Arthritis Res Ther. 2012 Jun 14;14(3):R145. doi: 10.1186/ar3879 (PMC3446529; doi:10.1186/ar3879)

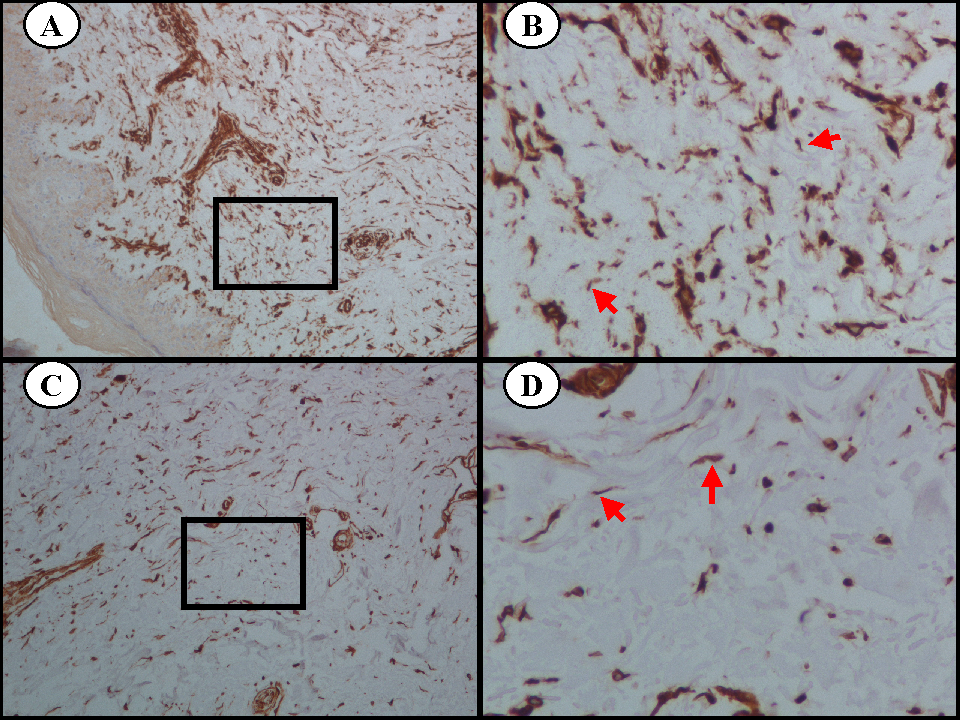

Supplement: Additional file 1 — Phosphorylated PDGFRβ expression in skin biopsies from Patient 1 (rituximab treated). A and B, biopsy taken at baseline; C and D, biopsy taken at 6 months. B and D, higher magnification of the areas included in the boxes in A and C, respectively. Red arrows indicate presence of spindle-like cells that express phospho-PDGFRβ. Streptavidin-biotin peroxidase: A and C, ×100; B and D, ×400. [file ar3879-S1.TIFF]

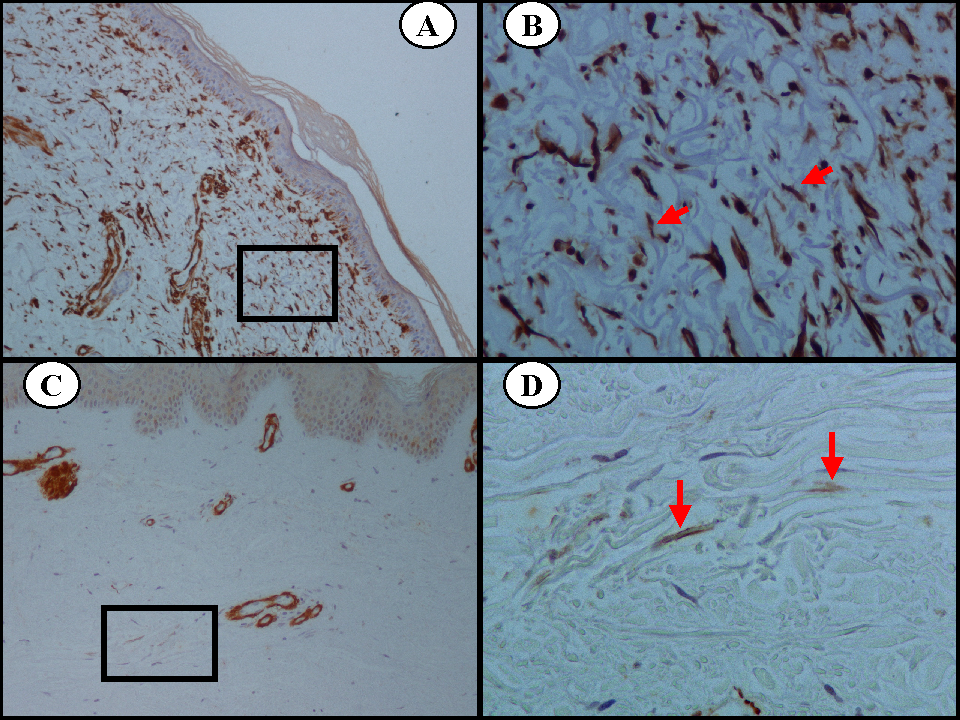

Supplement: Additional file 2 — Phosphorylated PDGFRβ expression in skin biopsies from Patient 2 (rituximab treated). A and B, biopsy taken at baseline; C and D, biopsy taken at 6 months. B and D, higher magnification of the areas included in the boxes in A and C, respectively. Red arrows indicate presence of spindle-like cells that express phospho-PDGFRβ. Streptavidin-biotin peroxidase: A and C, ×100; B and D, ×400. [file ar3879-S2.TIFF]

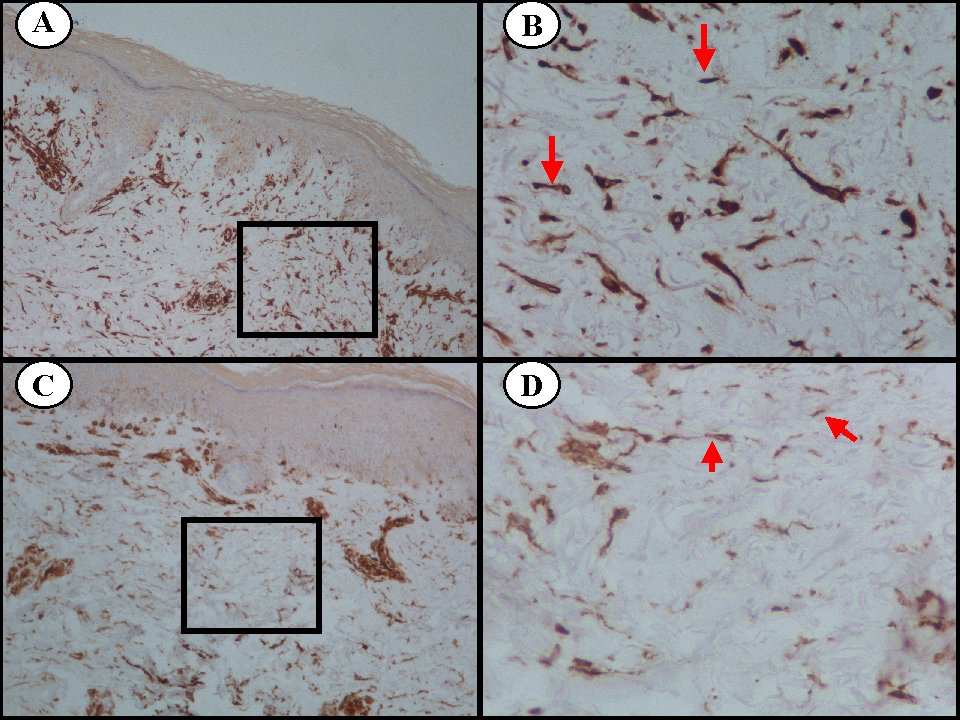

Supplement: Additional file 3 — Phosphorylated PDGFRβ expression in skin biopsies from Patient 3 (rituximab treated). A and B, biopsy taken at baseline; C and D, biopsy taken at 6 months. B and D, higher magnification of the areas included in the boxes in A and C, respectively. Red arrows indicate presence of spindle-like cells that express phospho-PDGFRβ. Streptavidin-biotin peroxidase: A and C, ×100; B and D, ×400. [file ar3879-S3.TIFF]

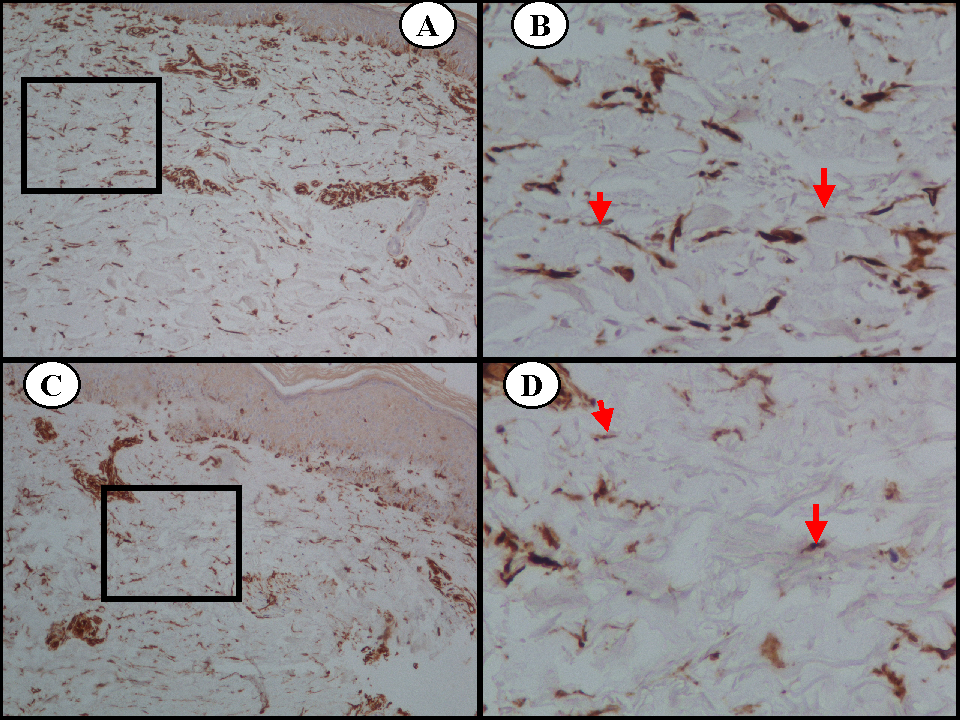

Supplement: Additional file 4 — Phosphorylated PDGFRβ expression in skin biopsies from Patient 4 (rituximab treated). A and B, biopsy taken at baseline; C and D, biopsy taken at 6 months. B and D, higher magnification of the areas included in the boxes in A and C, respectively. Red arrows indicate presence of spindle-like cells that express phospho-PDGFRβ. Streptavidin-biotin peroxidase: A and C, ×100; B and D, ×400. [file ar3879-S4.TIFF]

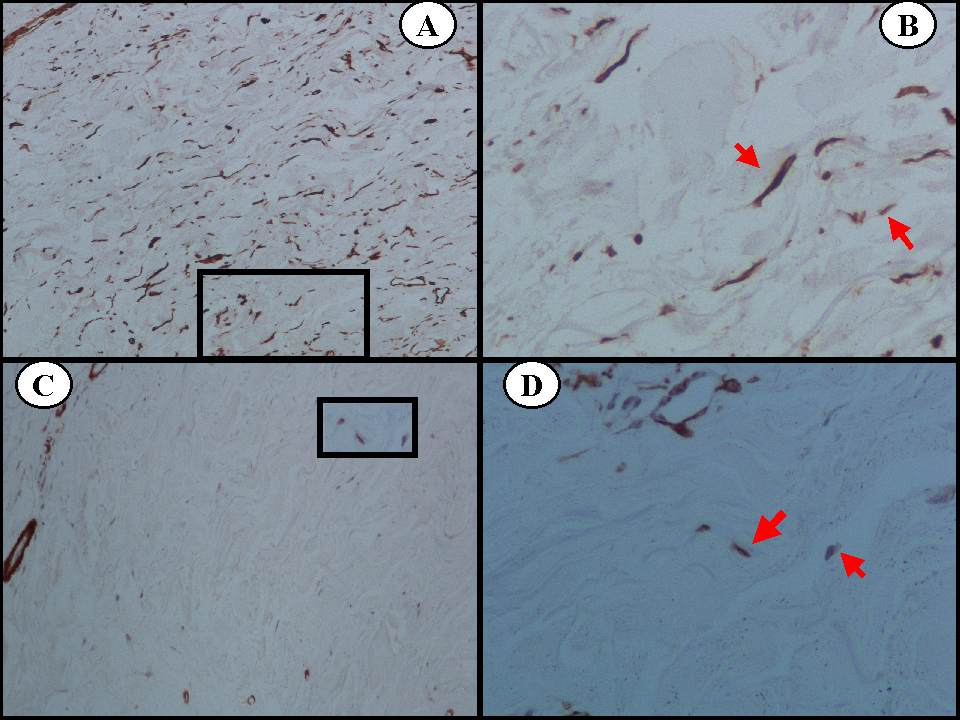

Supplement: Additional file 5 — Phosphorylated PDGFRβ expression in skin biopsies from Patient 5 (rituximab treated). A and B, biopsy taken at baseline; C and D, biopsy taken at 6 months. B and D, higher magnification of the areas included in the boxes in A and C, respectively. Red arrows indicate presence of spindle-like cells that express phospho-PDGFRβ. Streptavidin-biotin peroxidase: A and C, ×100; B and D, ×400. [file ar3879-S5.TIFF]

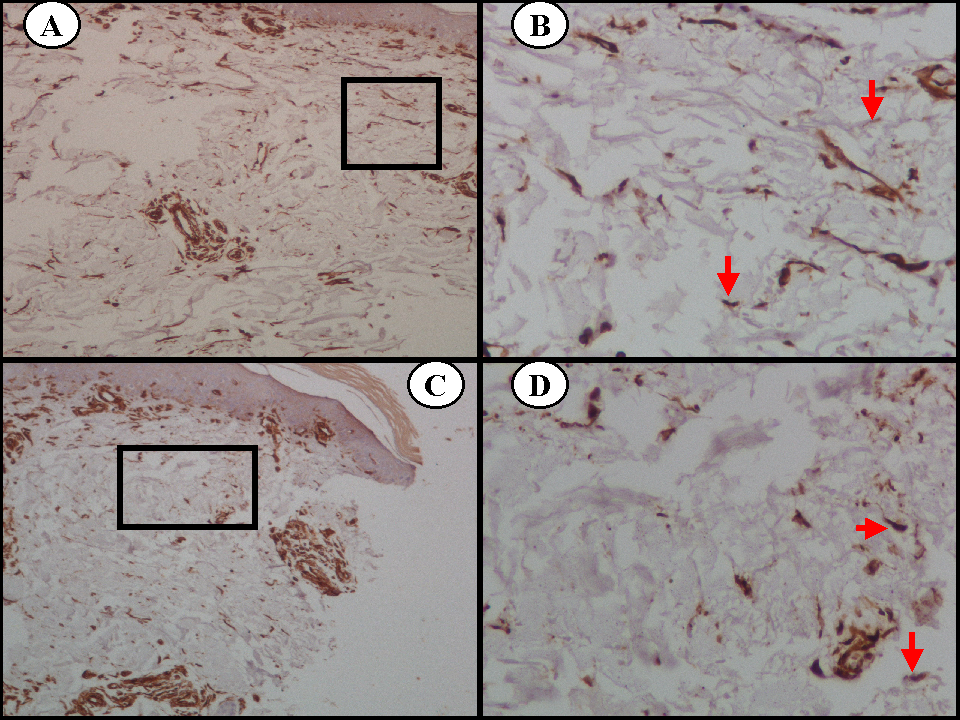

Supplement: Additional file 6 — Phosphorylated PDGFRβ expression in skin biopsies from Patient 6 (rituximab treated). A and B, biopsy taken at baseline; C and D, biopsy taken at 6 months. B and D, higher magnification of the areas included in the boxes in A and C, respectively. Red arrows indicate presence of spindle-like cells that express phospho-PDGFRβ. Streptavidin-biotin peroxidase: A and C, ×100; B and D, ×400. [file ar3879-S6.TIFF]

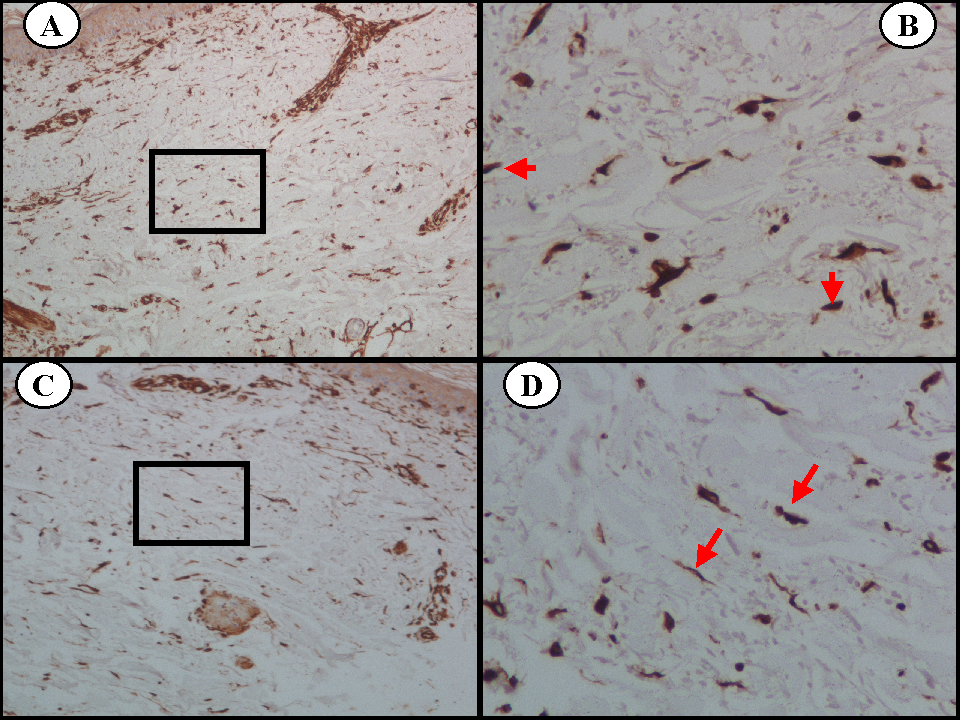

Supplement: Additional file 7 — Phosphorylated PDGFRβ expression in skin biopsies from Patient 7 (rituximab treated). A and B, biopsy taken at baseline; C and D, biopsy taken at 6 months. B and D, higher magnification of the areas included in the boxes in A and C, respectively. Red arrows indicate presence of spindle-like cells that express phospho-PDGFRβ. Streptavidin-biotin peroxidase: A and C, ×100; B and D, ×400. [file ar3879-S7.TIFF]

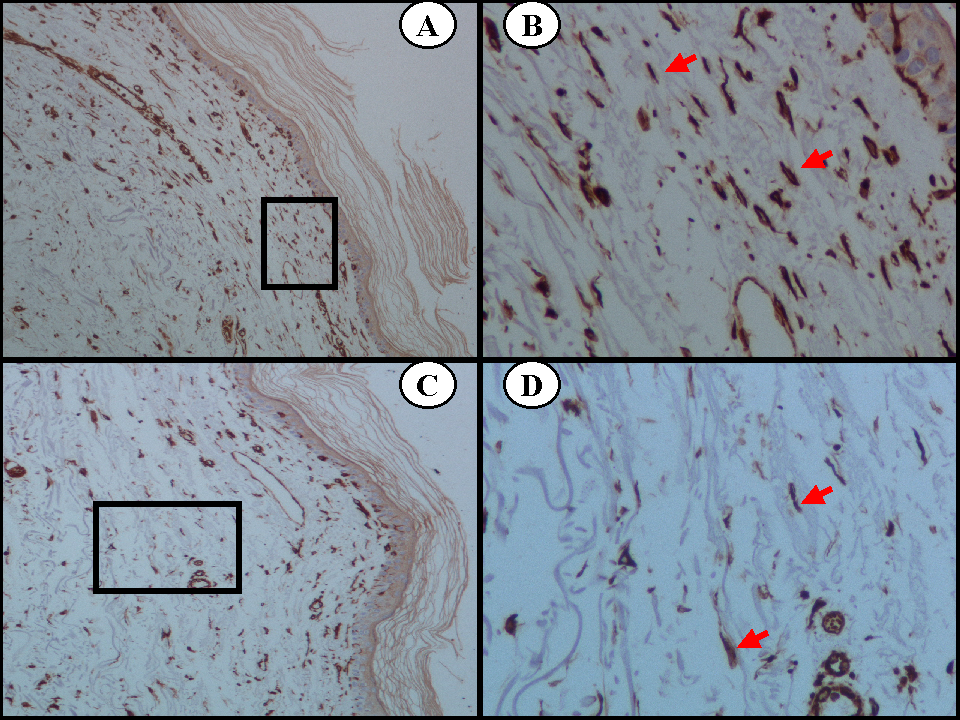

Supplement: Additional file 8 — Phosphorylated PDGFRβ expression in skin biopsies from Patient 8 (rituximab treated). A and B, biopsy taken at baseline; C and D, biopsy taken at 6 months. B and D, higher magnification of the areas included in the boxes in A and C, respectively. Red arrows indicate presence of spindle-like cells that express phospho-PDGFRβ. Streptavidin-biotin peroxidase: A and C, ×100; B and D, ×400. [file ar3879-S8.TIFF]

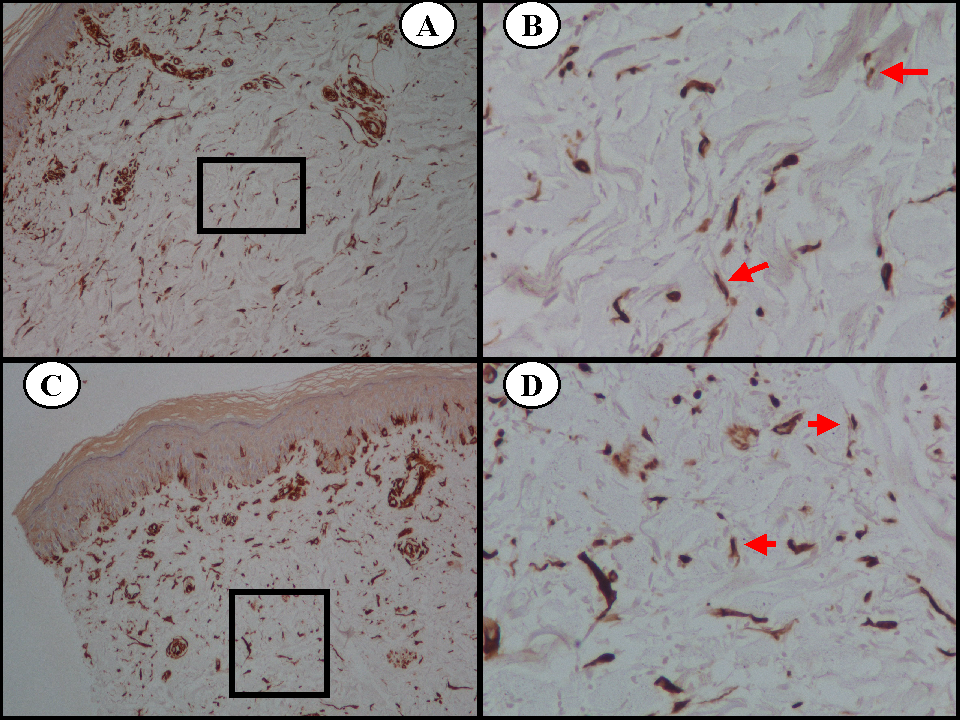

Supplement: Additional file 9 — Phosphorylated PDGFRβ expression in skin biopsies from control Patient 1. A and B, biopsy taken at baseline; C and D, biopsy taken at 6 months. B and D, higher magnification of the areas included in the boxes in A and C, respectively. Red arrows indicate presence of spindle-like cells that express phospho-PDGFRβ. Streptavidin-biotin peroxidase: A and C, ×100; B and D, ×400. [file ar3879-S9.TIFF]

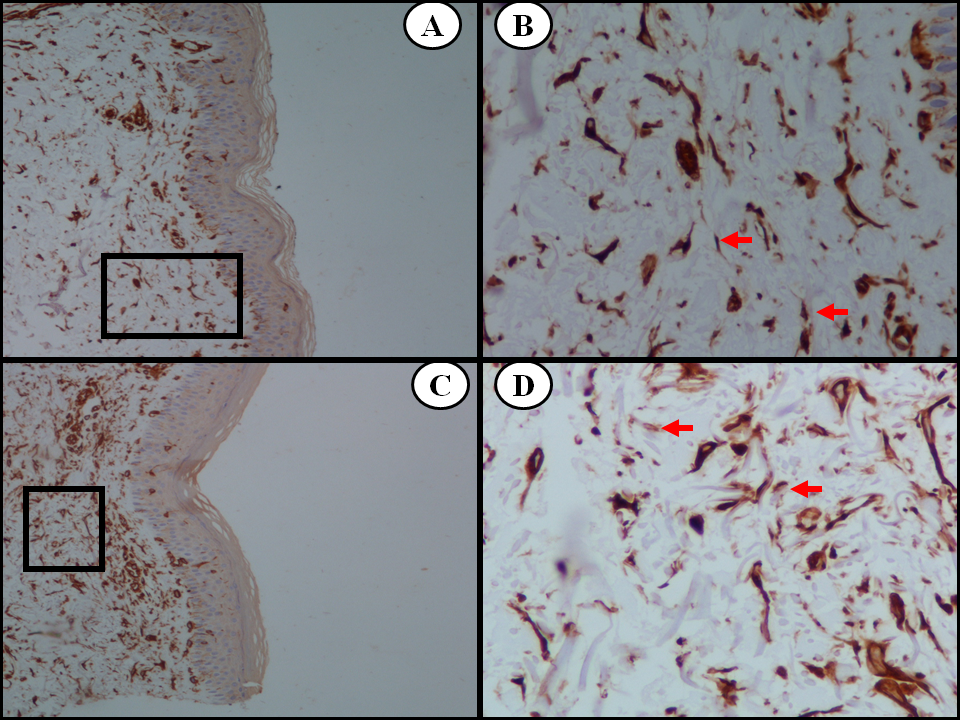

Supplement: Additional file 10 — Phosphorylated PDGFRβ expression in skin biopsies from control Patient 2. A and B, biopsy taken at baseline; C and D, biopsy taken at 6 months. B and D, higher magnification of the areas included in the boxes in A and C, respectively. Red arrows indicate presence of spindle-like cells that express phospho-PDGFRβ. Streptavidin-biotin peroxidase: A and C, ×100; B and D, ×400. [file ar3879-S10.TIFF]

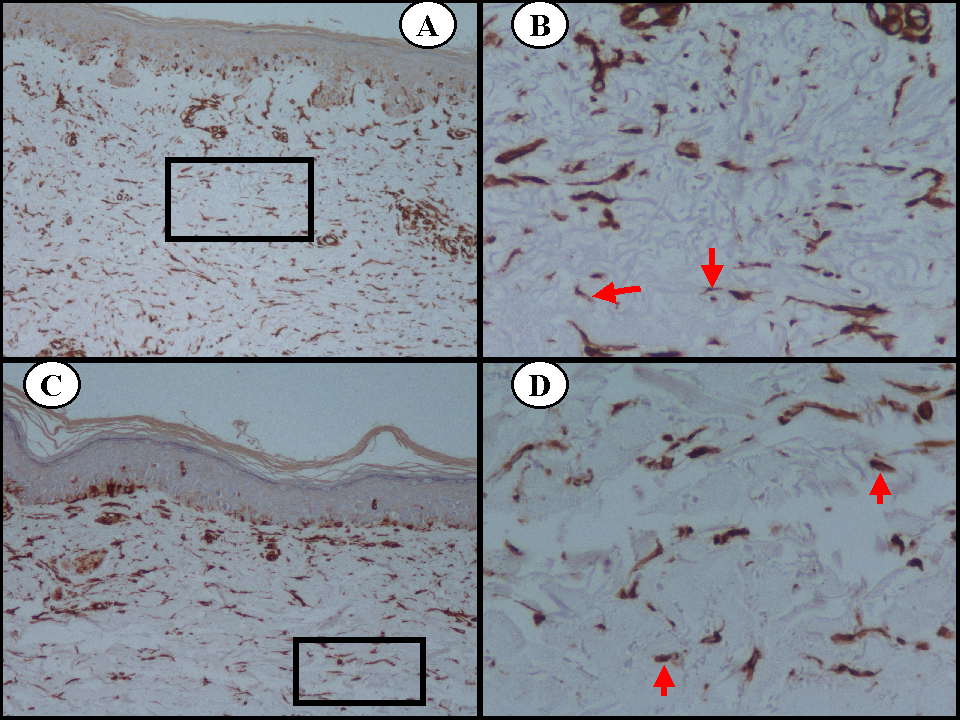

Supplement: Additional file 11 — Phosphorylated PDGFRβ expression in skin biopsies from control Patient 3. A and B, biopsy taken at baseline; C and D, biopsy taken at 6 months. B and D, higher magnification of the areas included in the boxes in A and C, respectively. Red arrows indicate presence of spindle-like cells that express phospho-PDGFRβ. Streptavidin-biotin peroxidase: A and C, ×100; B and D, ×400. [file ar3879-S11.TIFF]
